# Targeting PPAR-Regulated Pathways to Treat Cholestatic Liver Diseases: Novel Applications of Liquid Biopsies

**DOI:** 10.3390/cells15161442

**Published:** 2026-08-11

**Authors:** Colleen M. Hayes, Daniella R. Cross, Brahim Achour, Nisanne S. Ghonem

**Affiliations:** Department of Biomedical and Pharmaceutical Sciences, College of Pharmacy, University of Rhode Island, Avedisian Hall #395M, 7 Greenhouse Road, Kingston, RI 02881, USAachour@uri.edu (B.A.)

**Keywords:** cholestatic liver diseases, peroxisome proliferator-activated receptor, liquid biopsy, RNAseq, extracellular vesicles

## Abstract

Primary biliary cholangitis (PBC) and primary sclerosing cholangitis (PSC) are chronic cholestatic liver diseases with limited therapeutic options. First-line therapy for PBC is ursodeoxycholic acid, although up to 40% of patients respond incompletely, and there is no effective therapy for PSC. Newer peroxisome proliferator-activated receptor (PPAR) agonists, e.g., seladelpar and elafibranor, received accelerated FDA approval as second-line treatments for PBC, and additional studies of PPAR agonists for PSC are underway. PPAR agonists have varying affinities for the PPAR isoforms (α, δ, γ), and the functional effects of isoform activation in humans are less known. Interindividual PPAR isoform expression varies across diseases and traditionally required invasive tissue biopsies for evaluation. Newer experimental approaches, such as extracellular vesicles (EVs) from liquid biopsies, can be used to characterize individual gene expression as an alternative (to tissue biopsy). The transcriptomic profiling of EVs uniquely allows for the quantification of coding and non-coding RNA transcripts, which may be used to study pathways relevant to PPAR expression and regulation and to identify biomarkers of treatment response to PPAR agonists in cholestasis. This review explores the application(s) of liquid biopsy-derived EVs for the identification of PPAR regulated pathways and its potential role in the treatment of PBC and PSC.

## 1. Introduction

Primary biliary cholangitis (PBC) and primary sclerosing cholangitis (PSC) are autoimmune-mediated cholestatic liver diseases that are characterized by the accumulation of bile acids in the liver due to impaired bile flow [1]. Without effective therapy, disease progression can lead to inflammation, liver damage, and eventual liver failure [2]. First-line therapy for PBC is ursodeoxycholic acid (UDCA); however, up to 40% of people with PBC respond incompletely to UDCA [3], and there is no U.S. Food and Drug Administration (FDA)-approved therapy for PSC. Recent clinical investigations into peroxisome proliferator-activated receptor (PPAR) agonists as anti-cholestatic therapies have led to the accelerated FDA approvals of elafibranor (Iqirvo^®^) and seladelpar (Livdelzi^®^) as second-line therapies for PBC, i.e., refractory PBC, or for those experiencing a sub-therapeutic response to UDCA.

Therapeutic benefits of PPAR agonists as second-line therapies for PBC are supported by over a decade of clinical studies [4]. PPAR agonists exhibit variable selectivity for PPAR isoforms (α, δ, γ), resulting in differential regulation of genes and pathways, particularly those involved in bile acid synthesis and metabolism [5,6,7,8], fibrosis [9,10,11,12], inflammation [13], and modulating immune cell function [14,15]. Through overlapping but distinct mechanisms, these complementary effects contribute to their therapeutic efficacy in treating cholestatic liver diseases, while isoform-specific mechanisms may contribute, in part, to the differing therapeutic profiles and variability in response observed with selective or dual PPAR isoform agonists therapy in chronic cholestatic liver diseases.

Efficacy measurements of PPAR treatment for refractory PBC include surrogate clinical or biochemical endpoints, e.g., serum alkaline phosphatase (ALP), albeit a nonspecific measure of liver injury that does not indicate the treatment’s influence on pathways relevant to pharmacologic response or disease progression. Despite this, serum ALP is widely used as an endpoint in clinical studies of cholestasis, though ALP levels may spontaneously fluctuate in PSC [16,17] and may not necessarily reflect disease development or progression [18], thus emphasizing the need for more comprehensive biomarkers of efficacy.

The potency and selectivity of PPAR agonists for the individual PPAR isoforms [4] have been characterized by cell-based transactivation assays [19]; however, the extent to which PPAR agonists activate and modulate each isoform in humans is less known. Traditionally, elucidating these effects requires invasive tissue biopsies, which are limited in accessibility and availability, e.g., for research purposes. To overcome these limitations, recent developments have shifted towards the application of liquid biopsy approaches, which involve the extraction and analysis of extracellular vesicles (EVs) derived from biofluids [20,21], e.g., blood or urine, to characterize pathways relevant to drug disposition and response. EVs are lipid bilayer-enclosed vesicles released from multiple cell types and include exosomes (50–150 nm in diameter), microvesicles (100–1000 nm), and apoptotic bodies (50–5000 nm) [22]. EVs can contain diverse molecular biomarkers that include RNA, DNA, proteins, lipids, and other metabolites from the cells of origin [23]. The circulating RNA includes coding RNA (mRNA) and non-coding RNA (microRNA (miRNA), long non-coding RNA (lncRNA), etc.), in addition to proteins extracted from EVs [22,23]. The composition of EVs is dependent on the biologic fluid from which they are derived [22,23], and EV shedding by cells is affected by several factors, including disease and age [24]. EV-derived RNA can be used to characterize variability in the expression of liver-specific markers and therapeutic targets, including key enzymes, transporters, and transcription factors relevant to drug metabolism and disposition [21,25]. Thus, EVs isolated from liquid biopsy samples offer a minimally invasive and a more widely accessible alternative to tissue biopsy, while also preserving the ability to quantify changes in gene expression [21,26].

Additionally, EV extraction uniquely allows for the quantification of RNA transcripts as the membrane-bound vesicles protect against RNA degradation, allowing for over 20,000 human gene transcripts to be sequenced [21]. Recently, Achour et al. [24] observed that RNA transcripts measured in plasma-derived EVs correlate with in vivo activity after a suitable normalization to liver shedding of EVs into the bloodstream, particularly for cytochrome P450 enzymes and P-glycoprotein, thereby demonstrating the potential clinical application(s) of quantitative liquid biopsy measurements for liver diseases.

## 2. Methods

Literature searches were conducted in databases, such as PubMed and Google Scholar. Search terms including, but not limited to, “liquid biopsy”, “PPAR”, “cholestasis”, “primary biliary cholangitis”, “primary sclerosing cholangitis”, “RNA sequencing” (RNAseq), “transcriptomics”, “proteomics”, and “non-coding RNA” were used. The primary topics of interest were cholestatic liver disease, liquid biopsy, extracellular vesicles, transcriptomic and proteomic analyses, and non-coding RNAs relevant to PPAR expression. Articles that did not describe transcriptomic or proteomic analyses of RNA or protein extracted from extracellular vesicles were excluded. For the interaction between non-coding RNAs and PPAR, articles were selected if they characterized the interaction between PPAR and a specific non-coding RNA or if they demonstrated an association between PPAR and non-coding RNA expression using clinical samples, with a focus on patients with liver disease.

## 3. EV Analysis: Transcriptomic or Proteomic Profiling

Selective collection of EVs originating from a specific organ, e.g., liver, is a major EV sample preparation challenge. Published methods tend to either collect all released vesicles, e.g., with resin-based precipitation or ultracentrifugation (high recovery, low selectivity), while applying a mathematical correction for liver shedding and targeting liver-enriched genes [21], or to alternatively enrich liver-derived EVs using immunoprecipitation, e.g., against asialoglycoprotein receptor 1, a liver-specific membrane surface marker (low recovery, high selectivity) [27], followed by analysis of RNA and protein content using proteomic or transcriptomic methods. To date, there is no available method that can achieve both high recovery and highly selective EV profiles from specific tissues, and the choice of the sample preparation method and downstream analysis is generally determined by the intended applications, available instrumentation, and budget restrictions.

Transcriptomic and proteomic analyses of EVs both characterize disease-associated molecular pathways, though each approach has distinct advantages and limitations. Transcriptomics uniquely quantifies the whole transcriptome, including gene transcripts present at low abundance, monitored with suitable amplification of RNA targets, in contrast to the smaller subset that is both transcribed and translated into proteins in a specific tissue type in the case of proteomics. Nevertheless, mRNA transcripts do not necessarily correlate with protein levels [28] and, although proteomic approaches are restricted to protein expression alone, they provide information on the post-transcriptional modifications of proteins [29]. However, proteomic studies are often limited by mass spectrometry sensitivity and the detection of low-abundance proteins and, therefore, may fail to detect biologically relevant low-abundance proteins [29]. Our recent global proteomics analysis of healthy plasma-derived EVs quantified a relatively narrow range of proteins and did not yield informative data on drug disposition pathways [26]. We found that highly abundant serum proteins, e.g., albumin and immunoglobulins present as contaminants of the samples even after extraction of EVs from plasma, dominated the mass spectrometric profile, whereas transcriptomic analyses of plasma-derived EVs were not affected by this limitation [26]. It should be noted that protein depletion steps suitably applied with plasma proteomics can also be used with EVs but may affect the yield and distribution of plasma-derived EVs. While the findings suggest that transcriptomic approaches may be more practical than proteomic analyses for the study of plasma-derived EVs, larger sample volumes and more sensitive, higher-capacity mass spectrometry instruments are suggested to circumvent the low-abundance limitation of EV proteomics.

Transcriptomic approaches also provide insight into the expression of non-coding genes, e.g., lncRNA, miRNA, and circular RNAs, which influence biological processes and could be potential biomarkers of disease state(s) or therapeutic response. For example, whole transcriptomic sequencing of liver tissue from people with PBC and autoimmune hepatitis variant syndrome identified differentially expressed mRNAs, lncRNAs, circular RNAs, and miRNAs among patients who responded to therapy [30]. In particular, non-coding RNAs have been identified as potential diagnostic or prognostic markers in PSC [31], as well as in other liver diseases, including metabolic dysfunction-associated steatohepatitis (MASH)/metabolic dysfunction-associated fatty liver disease (MASLD) [29], cholangiocarcinoma (CCA) [31], hepatocellular carcinoma (HCC) [32], and biliary tract cancer [33], thereby demonstrating the importance of non-coding RNA molecules.

## 4. Role of EVs in Cholestatic Liver Diseases

**Figure 1:**
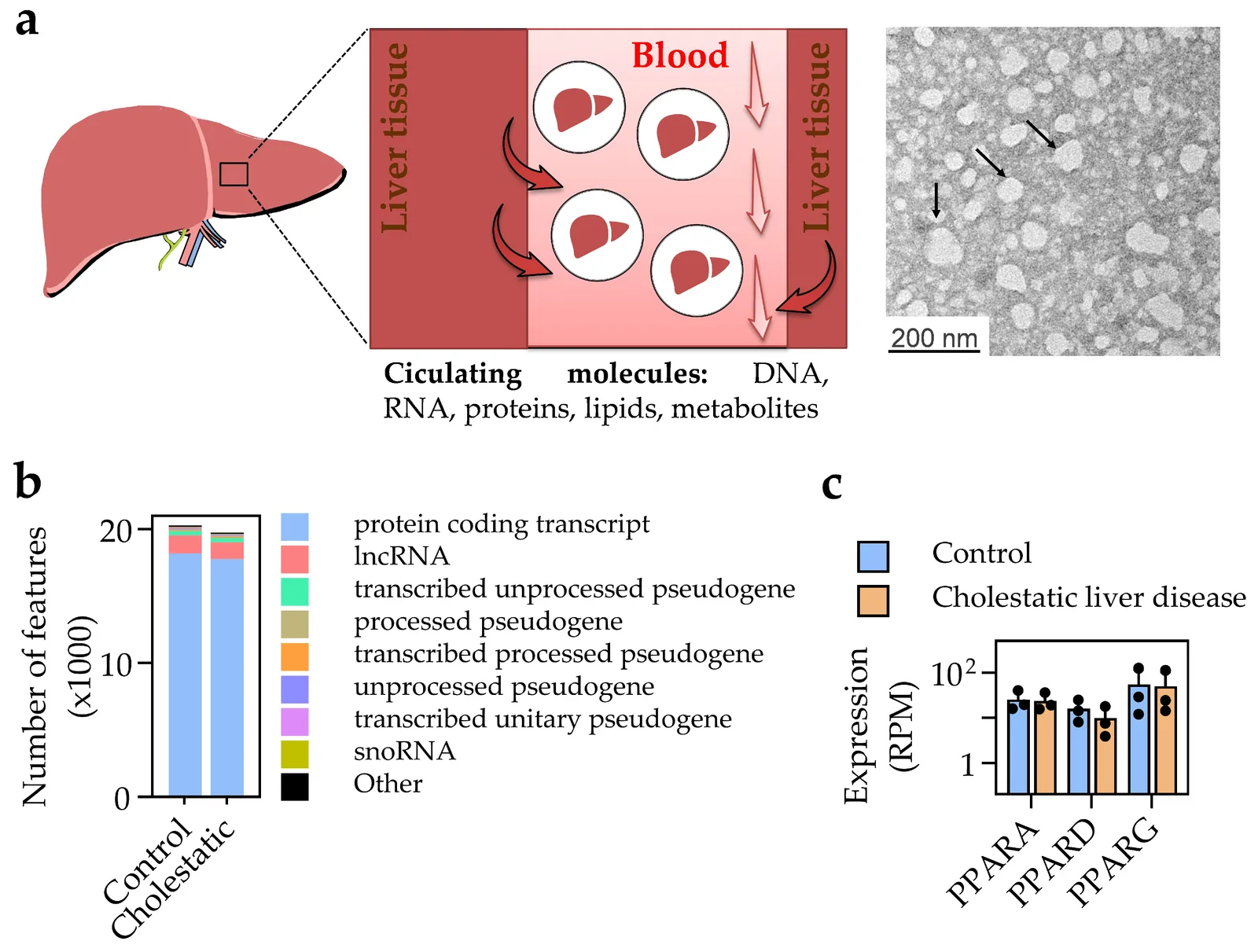
Feasibility of monitoring the expression of peroxisome proliferator-activated receptor (PPAR) genes in plasma-derived extracellular vesicles (EVs) using RNA sequencing (RNAseq). (**a**) EVs are released from different tissue types, particularly the liver, which is highly perfused and receives approximately a quarter of the total cardiac output through the portal vein and hepatic artery. EVs contain a snapshot of the molecular composition (RNA, DNA, and metabolites) of their tissue of origin at the time of shedding; (**b**) RNA isolated from EVs allows monitoring of coding and non-coding RNA from >20,000 genes in disease and control samples; (**c**) proof-of-concept RNAseq analysis confirms the feasibility of monitoring changes in PPAR expression in cholestatic liver disease [26]; lncRNA: long non-coding RNA; snoRNA: small nucleolar RNA; PPARA/D/G: peroxisome proliferator-activated receptor α/δ/γ; RPM: reads per million.

EVs sampled by liquid biopsy can be used to establish diagnostic markers that are specific to disease states and/or monitor the expression of genes using RNAseq in liver diseases. Recently, we demonstrated a proof-of-concept application of a liquid biopsy-RNAseq method to monitor the expression of more than 500 genes relevant to drug disposition in adult cholestatic liver disease (Figure 1) [26]. These findings are in line with our previous work, which demonstrates the same capacity in other disease states, including liver cancer [21], cardiovascular disease [24], and renal impairment [34]. While the small sample size of our cholestatic liver disease cohort may limit clinical interpretations of the data, this study supports the feasibility of transcriptomic profiling of over 20,000 genes, including protein-coding genes, lncRNA, miRNA, antisense RNA, and druggable targets [26]. Additional analyses of EVs obtained from liquid biopsy samples of PBC and PSC are summarized below.

### 4.1. Role of EVs in PBC: Transcriptomic and Proteomic Profiling

In PBC, transcriptomic analyses of EVs derived from plasma and serum have identified differentially expressed miRNAs between patients with PBC and healthy individuals. A miRNA expression array analysis of plasma-derived EVs identified nine significantly up-regulated and nine significantly downregulated miRNAs in PBC compared to healthy individuals [35]. The levels of miRNA-451a and miRNA-642a-3p were also significantly higher in a larger cohort of PBC; however, none of the evaluated exosomal miRNAs correlated with disease severity in this cohort [35]. Interestingly, miRNA-451 expression was significantly downregulated in patients with HCC compared to healthy individuals, and miRNA-451 inhibited the inflammatory markers IL-6, tumor necrosis factor α, and IL-1β, in vitro [36], suggesting that miRNA-451 protects against inflammation. Likewise, miRNA-451a levels were significantly decreased with disease progression of hepatitis C virus-infected HCC patients, and miRNA-451a improved lipid metabolism in vitro [37], suggesting that miRNA-451a possesses anti-inflammatory and metabolic regulatory functions in liver disease. Whether the increased expression of miRNA-451a in EVs from patients with PBC reflects a protective response is unknown. Alternatively, miRNA-642a-3p exerts tumor promotive properties, e.g., cancer cell migration and invasion in vitro, and miRNA-642a-3p knockdown inhibited tumor growth in vivo [38], though its role in PBC is unclear, and further analyses are needed.

Another RNAseq analysis of serum-derived exosomes identified 263 differentially expressed miRNAs in patients with PBC compared to healthy controls [39]. Interestingly, exosomal miR-122-5p levels were significantly increased in PBC, which positively correlate with serum levels of the pro-inflammatory cytokines, e.g., IL-6, IL-12, transforming growth factor-β1, and interferon-γ [39], as well as with the markers of injury, including serum ALP, ALT, AST, direct bilirubin, and γ-glutamyl transpeptidase [40]. Despite its modest diagnostic sensitivity (area under the receiver operating characteristic (AUC) curve of 0.701), miR-122-5p may serve as a diagnostic predictor of PBC in combination with other indicators [40]. Additionally, while elevated miR-122-5p is associated with greater biochemical evidence of liver injury, experimental injection of exosomes overexpressing miR-122-5p reduced hepatic portal inflammation, bile duct damage, and fibrosis in a murine model of PBC [40]. Likewise, miR-122-5p overexpression in intrahepatic biliary epithelial cells promoted proliferation while inhibiting apoptosis, epithelial-mesenchymal transition, and the expression of fibrosis-associated markers in vitro [40]. These findings suggest that elevated miR-122-5p may represent an adaptive response to liver injury, though additional studies are necessary. Importantly, studies also indicate that miR-122-5p may influence PPAR signaling through direct repression of PPARα [41] and PPARδ [42], suggesting that increased miR-122-5p could alter response to PPAR therapy; further studies are also needed to clarify this.

Collectively, these transcriptomic studies indicate that EV-derived miRNAs in PBC are associated with pathways involved in inflammation and liver injury. Most notably, miR-122-5p expression correlates with inflammatory markers and biochemical markers of liver injury in PBC and may influence PPAR expression. However, the biological significance of other miRNAs, e.g., miR-451a and miR-642a-3p, in cholestasis, and their connection to PPAR signaling are currently unknown. There are currently no available proteomic analyses of EVs in PBC.

### 4.2. Role of EVs in PSC: Transcriptomic and Proteomic Profiling

In PSC, transcriptomic and proteomic analyses of EVs extracted from serum [31,43,44,45] and urine [31] have identified several candidate biomarkers that could serve as non-invasive and unique diagnostic markers. Transcriptomic analyses of serum and urine-derived EVs from PSC patients revealed several mRNAs and non-coding RNAs (miRNA, lncRNA, small nucleolar RNA, pseudogenes), with high diagnostic values for PSC compared to ulcerative colitis and healthy individuals, and with candidate transcripts differing between serum and urine [31]. Despite the high diagnostic potential for PSC, it is critical to evaluate the specificity of the RNA transcripts as potential biomarkers in distinguishing between PSC and other disease states, such as CCA. This is particularly important, as PSC is a risk factor for CCA, and there is a lack of noninvasive methods to accurately distinguish these disease states [31,43]. Indeed, several coding and non-coding RNAs were identified as potential biomarkers for the differential diagnoses of CCA and PSC in both serum and urine-derived EVs, with AUCs up to 1.000 [31], demonstrating the high differential diagnostic potential of RNA molecules.

In addition to transcriptomic profiling, proteomic analyses of serum-derived EVs have also been performed in PSC. Proteomic analysis of EVs isolated from the serum of PSC patients and healthy controls revealed 161 differentially expressed proteins, with ficolin-1, neprilysin, and aminopeptidase N proteins having the best diagnostic capacity between PSC and healthy individuals, though aminopeptidase N also had high diagnostic capacity between CCA and healthy individuals [44]. Although pathway enrichment analysis was not reported, several of the identified proteins have established roles in innate immunity and inflammatory signaling, processes which are central to PSC pathogenesis [46]. For example, ficolin-1 and aminopeptidase N are potential therapeutic targets for autoimmune [47] and inflammatory [48] disorders, respectively, as ficolin-1 is a pattern-recognition molecule that activates the lectin complement pathway, and aminopeptidase N activates immune cells and pro-inflammatory cytokines. In addition, 10 proteins with high differential diagnostic capacity between CCA and PSC were identified, and the diagnostic capacities of these proteins changed when factoring in the stages of CCA [44]. These data demonstrate the importance of evaluating the expression of candidate biomarkers among similar disease states and stages to ensure accuracy and specificity.

Additional proteomic analyses of serum-derived EVs from patients with varying stages of isolated PSC, CCA, or overlapping diagnoses revealed distinct protein profiles between each group [43]. The identified candidate biomarkers were also detected in whole serum using enzyme-linked immunosorbent assay, thereby demonstrating their clinical translatability [43]. Interestingly, some of the candidate EV protein biomarkers were nearly exclusively expressed in hepatobiliary tissues [43]. Lastly, proteomic analysis of serum-derived EVs from PSC and healthy states identified 282 differentially expressed proteins, with the top upregulated proteins in PSC identified as Cystatin-S, IL-13 Ra1, CD83, IL-1β, and endothelial monocyte-activating polypeptide-II [45]. Notably, several of these proteins are involved in biological processes relevant to the pathogenesis of PSC, including inflammation and fibrogenesis. For example, IL-13 Ra1 mediates IL-13 signaling, a pathway which promotes liver fibrogenesis [49]. Consistent with this biological role, IL-13 Ra1, the most unique marker in EVs from PSC patients vs. healthy, was strongly associated with severe liver fibrosis in PSC [45]. IL-1β is a well-established pro-inflammatory cytokine that activates cultured murine hepatic stellate cells in vitro and contributes to steatosis, hepatocyte death, and fibrosis in mice [50]. Endothelial monocyte-activating polypeptide-II is a pro-inflammatory cytokine [51], and CD83 is a marker of activated immune cells [52], suggesting that differentially expressed EV-derived proteins reflect the inflammatory and profibrogenic characteristics of PSC and may provide mechanistic insight into disease progression following external validation. Furthermore, miRNA analysis of these EVs revealed 11 differentially expressed miRNAs, including the novel miR-4645-3p, whose biological functions are largely unknown, and miR-122-5p, which was also identified in PBC [40]. Although these candidate biomarkers require additional validation in larger cohorts of subjects, these findings support the use of liquid biopsy-derived EVs as a minimally invasive approach to identify potential biomarkers of PSC. Future studies that incorporate pathway enrichment analyses of EV-derived datasets may help to determine whether altered EV RNA or proteins are associated with metabolic pathways, including bile acid metabolism or PPAR-regulated signaling.

## 5. Role of PPAR(s) as Therapeutic Target(s) for Cholestatic Liver Diseases

Therapeutic effects of PPAR agonists are attributed to their preferential activation of PPAR isoforms (α, δ, γ), of which each ligand has dose-dependent isoform-selective potency [4]. Given the importance of PPAR activation, interindividual differences in endogenous PPAR expression have been hypothesized to contribute to variability in therapeutic response, although this relationship remains to be established in cholestatic liver disease. Indeed, pharmacogenomic studies indicate that genetic variations within the PPARα gene impact the response to the PPARα agonist fenofibrate, i.e., lipid and inflammatory marker responses in non-cholestatic patients [53,54], suggesting that patient-specific genetic variation within PPAR genes influences therapeutic response, although the contribution of endogenous PPAR expression is poorly understood.

Nevertheless, significant variations in endogenous PPAR expression have been described across liver disease stages and etiologies. For example, tissue biopsies demonstrate that PPARα mRNA [55] and protein [55,56] expression are significantly reduced in advanced-stage PBC patients undergoing liver transplantation [55] and in biliary atresia [56] compared to healthy individuals. Interestingly, PPARα mRNA levels are significantly increased in early-stage PBC compared to healthy individuals, after which, levels significantly decrease by advanced-stage [55], suggesting that the regulation of PPARα changes over the course of the disease, therefore raising the possibility that treatment response to PPAR therapy may also vary over time. In PSC, mRNA expression of PPARα is significantly increased [57], whereas in MASH, significantly decreased PPARα expression correlates with more severe steatosis, ballooning, and fibrosis [58]. Given these disease-specific and stage-dependent variations in PPAR expression, evaluating endogenous PPAR isoform expression and its modulation by PPAR agonists is of particular interest in cholestasis because a modest number of patients with refractory cholestatic disease do not achieve a therapeutic response to second-line treatment with PPAR agonists. For example, in our clinical studies of second-line treatment with fenofibrate in PBC and PSC, nearly 20% of patients did not achieve normalized ALP levels [59], and in recent phase III clinical studies of PPAR agonists in PBC, nearly 33–85% of patients treated with bezafibrate [60], seladelpar [61], or elafibranor [62] did not achieve normalized ALP levels. In PSC, serum ALP normalization was not achieved in over 80% of patients treated with elafibranor [63]. These data indicate that a sizeable proportion of patients with refractory cholestatic liver disease may not achieve sufficient clinical benefit from PPAR agonist treatment for reasons that are currently unclear. Although the mechanisms underlying this variability are unknown, differences in endogenous PPAR expression are a plausible biological contributor. As such, characterization of endogenous PPAR expression and its modulation by PPAR agonists may improve our understanding of interindividual variability in treatment response and, furthermore, may serve as a means to personalized treatment approaches, following rigorous clinical validation. Importantly, we have demonstrated the feasibility of quantifying the RNA expression of each PPAR isoform (and other relevant pathways) in our liquid biopsy-RNAseq method [26], which may overcome the need for more invasive procedures, e.g., tissue biopsy (Figure 1). However, while these findings establish the technical feasibility of this approach, their translational implications remain uncertain, as the clinical validity and utility of EV-based transcriptomic profiling in cholestasis have yet to be established. To date, there is no clinical evidence that EV-based transcriptomic signatures can reliably predict response to PPAR agonists in patients with cholestatic liver disease. Future prospective studies in large-scale, well-characterized cohorts, together with external validation, will be essential to determine the clinical applicability of this approach and whether it can meaningfully inform therapeutic decision-making or improve patient outcomes.

## 6. Effect of Non-Coding RNA on PPAR Isoform Expression

In addition to quantifying the RNA expression of PPAR isoforms, transcriptomic profiling of EVs also provides insight into the RNA expression of non-coding RNAs that regulate PPAR expression through direct and indirect mechanisms. The direct interactions between non-coding RNAs and PPAR isoforms are summarized in Table 1. In general, direct-acting non-coding RNAs downregulate PPAR protein expression by binding to the 3′ untranslated region (UTR) of PPARα [64,65,66,67,68], PPARδ [42], or PPARγ [69,70,71] mRNA. For example, the immunomodulator miR-21 binds to the 3′UTR of PPARα mRNA, thereby directly suppressing the formation of PPARα protein [64]. Interestingly, the expression of miR-21 is significantly elevated in the livers of early- and advanced-stage PBC by ~2- and 50-fold, respectively, compared to healthy individuals, and its levels significantly correlate with reduced PPARα mRNA expression in PBC patients [55]. Also, miR-21 is significantly increased in patients with PSC, though PPARα mRNA expression in this cohort was unchanged relative to healthy controls [55]. Furthermore, the expression of miR-21 is ~3-fold higher in the livers of patients with MASH compared to healthy controls [72], suggesting its clinical relevance in liver disease. Similarly, the expression of miR-155, an immunomodulatory non-coding RNA, is increased ~4- and 3.5-fold in the livers of patients with early- and late-stage PBC, respectively, and may contribute to PPARα downregulation in PBC [55]. In vitro data demonstrate that miR-155 significantly reduces the luciferase activity of 293T cells transfected with a PPARα 3′UTR construct [65], suggesting that miR-155 directly binds to the 3′UTR of PPARα. Interestingly, UDCA treatment significantly reduces miR-155 expression in HepG2 cells and cholangiocytes [55]. Although miR-21 and miR-155 are negatively correlated with PPARα expression in the liver of PBC subjects [55], these relationships were not observed in the peripheral blood mononuclear cells of patients with PBC [73].

The miRNA MIR20B is also significantly negatively correlated with PPARα mRNA expression in liver tissues of patients with MASLD, and luciferase assays indicate that MIR20B binds the 3′UTR of PPARα [66]. Meanwhile, MIR20B overexpression in HepG2 cells leads to significantly reduced mRNA levels of PPARα target genes, e.g., those involved in fatty acid β-oxidation and uptake [66], suggesting that MIR20B reduces PPARα activity. Additionally, miR-9 inhibits PPARα by binding its 3′UTR and significantly reducing PPARα mRNA and protein levels following miR-9 overexpression in SNU-449 [67] and HepG2 [68] cells, respectively. In addition, miR-9 expression is significantly increased (6.5-fold) and PPARα mRNA expression is significantly reduced (>2-fold) in the livers of HCC patients relative to controls, resulting in a negative correlation between PPARα mRNA and miR-9 expression [67].

PPARδ and PPARγ are also targets of non-coding RNAs. For example, miR-122-5p may promote inflammation through downregulation of PPARδ, as there is a putative binding site for miR-122-5p in the 3′-UTR of PPARδ, and miR-122-5p overexpression significantly reduces the luciferase activity of a PPARδ reporter gene in 293T transfected cells [42]. Additionally, miR-145 downregulates PPARγ expression in LX-2 cells transfected with a PPARγ 3′UTR fragment and an miR-145 mimic and significantly reduces PPARγ protein levels in cells transfected with an miR-145 mimic [69]. Importantly, miR-145 is significantly increased in the serum of patients with fibrosis, and PPARγ mRNA and protein levels are significantly decreased [69]. Furthermore, miR-130a also inhibits PPARγ by binding its 3′UTR and reduces PPARγ-mediated effects, e.g., reduced lipid droplet accumulation and triglyceride levels, in HepG2 and Huh-7 cells transfected with miR-130a mimics and treated with free fatty acids [70]. In patients with MASH, the expression of miR-130a-3p, the 3′ arm of miR-130a [74], is substantially reduced [75], and the expression of hepatic PPARγ mRNA (>2-fold) and protein (>1.5-fold) is significantly increased [76] relative to healthy livers. Moreover, miR-942 directly interacts with the 3′UTR of PPARγ as demonstrated by significantly reduced luciferase activity in transfected LX-2 cells, and decreased PPARγ mRNA and protein expression in cells overexpressing miR-942 [71]. Importantly, PPARγ expression is decreased in the liver tissues of patients with chronic hepatitis B and is significantly negatively correlated with miR-942 [71]. These studies demonstrate that all PPAR isoforms can be directly targeted by miRNAs.

**Table 1:** MicroRNAs (miRNAs) that directly inhibit peroxisome proliferator-activated receptor (PPAR) isoforms through binding to the 3′ untranslated region (UTR) of PPAR mRNA and their expression in liver diseases. Non-coding RNA expressions in patients with liver disease are reported relative to healthy controls, unless otherwise specified. * Fold-change approximated based on the source. n.s. not significant.

miRNA	Target PPAR Isoform(s) [Ref]	Non-Coding RNA Expression in Liver Diseases [Ref]
miR-21	PPARα [64]	PBC: ~2- * and 50-fold increase in hepatic expression in early-stage (*p* = 0.02) and late-stage PBC (*p* = 0.0001), respectively [55]; no change * in PBMC expression (n.s.) [73]. PSC: ~30-fold * increase in hepatic expression (*p* = 0.003) [55]; ~2-fold * decrease in PBMC expression (*p* = 0.02) [73]. MASLD: no change * in hepatic expression in bland steatosis (n.s.) [72]. MASH: ~3-fold * increase in hepatic expression (*p* < 0.01) [72].
miR-155	PPARα [65]	PBC: ~4- * and 3.5-fold increase in hepatic expression in early-stage (*p* = 0.0006) and late-stage PBC (*p* = 0.004), respectively [55]; no change * in PBMC expression (n.s.) [73]. PSC: ~2-fold * increase in hepatic expression (n.s.) [55]; ~2-fold * increase in PBMC (*p* = 0.03) [73].
MIR20B	PPARα	MASLD: ~3-fold * increase in hepatic expression in steatosis (*p* < 0.001) [66]. MASH: ~8-fold * increase in hepatic expression (*p* < 0.001) [66].
miR-9	PPARα [67,68]	HCC: 6.5-fold increase in hepatic tumor expression vs. adjacent non-tumor tissue (*p* < 0.001) [67].
miR-122-5p	PPARδ [42]	Unknown
miR-145	PPARγ [69]	Hepatic fibrosis: ~2-fold * increase in serum expression (*p* < 0.01) [69].
miR-130a	PPARγ [70]	MASH: major reduction in miR-130a-3p expression in liver tissue [75].
miR-942	PPARγ [71]	Chronic hepatitis B: greater hepatic expression in patients with F3 and F4 vs. F0 stage fibrosis (*p* < 0.01) [71].

Abbreviations: miRNA: microRNA; PPAR: peroxisome proliferator-activated receptor; UTR: untranslated region; PBC: primary biliary cholangitis; PBMC: peripheral blood mononuclear cell; PSC: primary sclerosing cholangitis; MASLD: metabolic dysfunction-associated fatty liver disease; MASH: metabolic dysfunction-associated steatohepatitis; HCC: hepatocellular carcinoma.

In addition to the direct impact on PPAR expression, non-coding RNAs may indirectly affect PPAR signaling. Indirect and uncharacterized interactions between non-coding RNAs and PPAR isoforms are summarized in Table 2. For example, non-coding RNAs may act as competitive endogenous RNAs that prevent miRNA-induced protein degradation by directly binding to and inhibiting miRNAs [69,70,77]. The lncRNA maternally expressed gene 3 (MEG3) prevents miR-145 from degrading PPARγ by competing with PPARγ for its miR-145 binding site [69]. Likewise, luciferase assays indicate that miR-145 binds to the 3′UTR of MEG3, and overexpression of MEG3 significantly reduces miR-145 expression in vitro [69]. Moreover, lncRNA H19 also indirectly upregulates PPARγ expression by binding to miR-130a, thereby preventing it from binding to and suppressing PPARγ [70]. Interestingly, H19 expression is significantly increased by >10-fold in the liver tissues of MASH patients compared to healthy controls [78]. Alternatively, the lncRNA highly upregulated in liver cancer (HULC) indirectly increases PPARα expression by promoting epigenetic modifications [68]; in vitro assays show that HULC promotes the methylation of CpG islands within the miR-9 promoter and significantly reduces its transcription dose-dependently, thereby preventing inhibition of PPARα by miR-9 and promoting lipogenesis through acyl-CoA synthetase long-chain 1, a PPARα target gene [68]. Interestingly, HULC expression significantly correlates with reduced miR-9 and increased PPARα mRNA expression in human HCC tissues, highlighting its clinical significance [68].

Metastasis-associated lung adenocarcinoma transcript 1 (MALAT1) is an lncRNA that influences both PPARα and PPARγ expression; in an in vitro model of MASLD, fatty acid treatment of HepG2 cells significantly reduces PPARα and increases PPARγ protein expression, and knockdown of MALAT1 reverses these changes to increase PPARα and reduce PPARγ protein expression toward control levels [77], whereas MALAT1 directly upregulates the transcription of the PPARγ promoter [79]. MALAT1 also acts as a competitive endogenous RNA to indirectly reduce PPARα expression by interacting with miR-206 and preventing miR-206 from inhibiting aryl hydrocarbon receptor nuclear translocator, a negative regulator of PPARα [77]. Importantly, MALAT1 and aryl hydrocarbon receptor nuclear translocator expression are significantly increased, and the expression of miR-206 and PPARα are decreased in liver tissues of patients with MASLD compared to healthy controls [77]. These data show that non-coding RNAs can regulate the expression of PPAR isoforms in an isoform-specific manner.

Although some of the mechanisms have yet to be elucidated, non-coding RNAs may also broadly modulate PPAR signaling. Liao et al. identified 827 differentially expressed lncRNAs within HCC tumor and healthy liver tissue, 13 of which were used to construct a prognostic signature for HCC [32]. Interestingly, functional enrichment analysis of protein-coding genes associated with these 13 prognostic lncRNAs revealed that PPAR signaling is functionally enriched in HCC [32]. Additionally, the significant increase of miR-675-3p expression in serum-derived exosomes of patients with HCC (relative to healthy individuals) is associated with poor prognosis, and functional enrichment analyses of exosomal RNA sequences identified PPAR signaling as a potential downstream target pathway of miR-675-3p [80]. Moreover, plasma-derived exosomal miRNAs from patients with MASLD or MASH were found to have significant positive (miR-122, miR-128) and negative (miR-200, miR-298, miR-342) correlations with plasma PPARγ levels [81]. The association of non-coding RNAs with PPAR expression demonstrates that the evaluation of non-coding RNA expression in EVs could be a useful tool to better understand how PPARs are expressed and how their expression affects disease severity.

**Table 2:** Non-coding RNAs that indirectly regulate peroxisome proliferator-activated receptor (PPAR) isoforms, and their expression in liver diseases. Long non-coding RNAs (lncRNAs) may indirectly regulate PPAR expression by acting as competitive endogenous RNAs, or through other regulatory mechanisms. For several non-coding RNAs, the molecular interaction with PPAR is not fully characterized, although some evidence suggests potential clinical relevance. Non-coding RNA expression in patients with liver disease are reported relative to healthy controls, unless otherwise specified. * Fold-change approximated based on the source; ** Interaction with PPARγ is not fully characterized. n.s. not significant.

Non-Coding RNA (*Type*)	Interaction with PPAR [Ref]	Non-Coding RNA Expression in Liver Diseases [Ref]
MEG3 (*lncRNA*)	Indirect activation of PPARγ through inhibition of miR-145 [69].	Hepatic fibrosis: ~2-fold * decrease in serum expression in fibrosis (*p* < 0.01) [69].
H19 (*lncRNA*)	Indirect activation of PPARγ through inhibition of miR-130a [70].	MASH: >10-fold increase in hepatic expression (*p* < 0.05) [78].
HULC (*lncRNA*)	Indirect activation of PPARα through epigenetic suppression of miR-9 [68].	HCC: 32.7-fold increase in hepatic expression vs. non-tumor liver tissue (*p* = 0.016) [82].
MALAT1 (*lncRNA*)	Indirect inhibition of PPARα through prevention of the miR-206 suppression of aryl hydrocarbon receptor nuclear translocator [77]; Direct interaction with PPARγ promoter to promote transcription [79].	MASLD: ~1.7-fold * increase in hepatic expression (*p* < 0.05) [77].
miR-675-3p (*miRNA*)	PPAR signaling is a potential downstream target pathway [80].	HCC: ~1.8-fold * increase in serum-derived exosomal expression (*p* < 0.001) [80].
miR-122, miR-128 (*miRNAs*)	** Positive correlation in plasma-derived exosomes of patients with MASLD/MASH and healthy controls (*p* < 0.001) [81].	*All refer to plasma-derived exosomal expression* [81]: *miR-122* MASLD: 1.5-fold increase (*p* < 0.05); MASH: 2.6-fold * increase (*p* < 0.05). *miR-128* MASLD: 1.1-fold * increase (n.s.); MASH: 1.7-fold increase (*p* < 0.05)
miR-200, miR-298, miR-342 (*miRNAs*)	** Negative correlation in plasma-derived exosomes of patients with MASLD/MASH and healthy controls (*p* < 0.001) [81].	*All refer to plasma-derived exosomal expression* [81]: *miR-200* MASLD: 1.1-fold * decrease (n.s.); MASH: 2-fold decrease (*p* < 0.05). *miR-298* MASLD: 1.3-fold decrease (*p* < 0.05); MASH: 2.7-fold * decrease (*p* < 0.05) *miR-342* MASLD: 1.3-fold decrease (*p* < 0.05); MASH: 1.9-fold * decrease (*p* < 0.05).

Abbreviations: lncRNA: long non-coding RNA; PPAR: peroxisome proliferator-activated receptor; MEG3: maternally expressed gene 3; MASH: metabolic dysfunction-associated steatohepatitis; HULC: highly upregulated in liver cancer; HCC: hepatocellular carcinoma; MALAT1: metastasis-associated lung adenocarcinoma transcript 1; MASLD: metabolic dysfunction-associated fatty liver disease; miRNA: microRNA.

## 7. Conclusions and Future Directions

This review provides an up-to-date overview of the role of EVs in chronic cholestatic liver diseases, namely PBC and PSC. Evidence from transcriptomic and proteomic analyses of EVs isolated from people with PBC and PSC, and the relative advantages of transcriptomic versus proteomic profiling have been summarized. In addition, the importance and feasibility of characterizing endogenous PPAR isoform expression in patients with cholestasis have been highlighted, including the influence of non-coding RNAs on PPAR signaling. Together, these insights support the potential of EV-based transcriptomic profiling as a valuable research tool to improve mechanistic understanding of PPAR agonist therapies and to inform the development of more individualized, precision medicine-driven treatment strategies for PBC and PSC, although additional studies are needed to validate its clinical utility and determine its role in routine clinical practice.

With two PPAR agonists recently approved by the FDA for second-line therapy in PBC, each having distinct selectivity and potency for individual PPAR isoforms, there is growing interest in distinguishing these agents based on PPAR isoform(s) activation and their corresponding pharmacologic effects. Currently, there is no standardized approach to guide clinical selection among the available PPAR agonists, highlighting the need for mechanistic and pharmacologic data to support a precision medicine approach. RNAseq of plasma-derived EVs may offer the necessary precision medicine approach to guide the treatment of cholestatic liver diseases. The declining cost of sequencing and wider availability of instrumentation should facilitate wider implementation of the technique. Furthermore, the RNA expression of non-coding and protein-coding RNAs relevant to PPAR expression, signaling, metabolism, and transcriptional regulation can be profiled. In addition, candidate biomarkers of disease state(s) may be identified to monitor disease progression over time. As large-scale transcriptomic datasets from patients with PBC and PSC become more readily available, future analyses may identify novel markers of biochemical response to PPAR agonists and support the development of evidence-based treatment algorithms, although current evidence for the clinical validity of such an approach is limited. While additional validation in independent cohorts and prospective clinical studies will be required, published findings highlight the potential of EV-based transcriptomic profiling to contribute to a more personalized approach to the management of cholestatic liver diseases.

In closing, advances in methodology, availability of technology and cost-reduction have been the driving factors for early implementation attempts of EV technology in clinical studies [24] and in drug development [83]. Wider implementation in clinical practice has been precluded thus far by several barriers, including technical standardization across laboratories, development of fit-for-purpose bioinformatics pipelines, reagent quality, capital cost minimization, and, ultimately, validation in large cohorts of patients, i.e., beyond proof-of-concept studies that have so far been reported [24,25,27]. Technical standardization of bioanalysis and bioinformatics will require large cross-laboratory efforts to produce validated guidelines, protocols, quality controls, and normalization standards, ensuring a benchmark for quality is established, similar to efforts by the Clinical Proteomic Tumor Analysis Consortium, part of the National Cancer Institute, in the cancer bioanalysis sphere [84]. Whereas the costs of multi-omics analyses at core facilities and commercially, particularly sequencing, have been on the decline over the past decade, capital cost related to acquiring and operating high-end sequencers and mass spectrometers remains high. This type of capital investment also includes training of personnel and quality assurance at different stages of analysis. In addition, clinical validation of EV technology is necessary to establish the appropriate applications and the limitations of the technique. Dedicated clinical studies may not be necessarily required, and this step can opportunistically be part of larger clinical trials and concerted industry efforts, where liquid biopsy measurements and other clinical endpoints are monitored in the same cohort(s) of patients [24,34]. Once established, these efforts should further support the application of liquid biopsy as a patient characterization tool for precision medicine.

## Data Availability

The RNAseq data used to generate Figure 1 are deposited on the NCBI GEO database under accession number GSE302189.
